# Factors affecting general sleep pattern and quality of sleep in pregnant women

**DOI:** 10.4274/tjod.22120

**Published:** 2015-03-15

**Authors:** Soner Ölmez, Hamit Sırrı Keten, Selçuk Kardaş, Fazıl Avcı, Ahmet Ferit Dalgacı, Salih Serin, Fatma Kardaş

**Affiliations:** 1 Kahramanmaraş Sütçü İmam University Faculty of Medicine, Department of Family Medicine, Kahramanmaraş, Turkey; 2 12 Şubat Community Health Center, Kahramanmaraş, Turkey; 3 Kahramanmaraş Sütçü İmam University Faculty of Medicine, Department of Psychiatry, Kahramanmaraş, Turkey; 4 Kahramanmaraş Sütçü İmam University Faculty of Medicine, Department of Obstetrics and Gynecology, Kahramanmaraş, Turkey; 5 Atatürk Training and Research Hospital, Clinic of Obstetrics and Gynecology, Ankara, Turkey; 6 Medipol University Faculty of Medicine, Department of Women’s Health and Birth Nurse, İstanbul, Turkey

**Keywords:** Pregnant, Sleep, epworth

## Abstract

**Objective:**

To investigate factors affecting general sleep pattern and sleep quality in pregnant women.

**Materialds and Methods:**

We assessed all pregnant women applied to Department of Obstetrics and Gynecology in Training and Research Hospital, School of Medicine, Kahramanmaraş Sütçü İmam University between 01 January 2014 and 01 March 2014. The participants were informed prior to the study and 100 pregnant women who gave their informed consent were included in the study. Questionnaires consisting sociodemographic characteristics, pregnancy history and the Epworth sleepiness scale were applied to the patients. Factors affecting general sleep pattern and sleep quality in pregnant women were compared.

**Results:**

The mean age of 100 pregnant women was 27.9 years (min=16, max=42). The mean gestational age of participants was found to be 24.8 weeks (min=5, max=40). In obstetric history, 9% of women previously had a stillbirth and also 25% of women previously had curettage performed. There were tobacco use in 6% of participants and 6% of patients previously had been to the hospital due to a sleep disorder. The mean excessive daytime sleepiness scale score of pregnant women were found to be 4.56. There were no significant difference among the patients regarding regular exercise (p=0.137), tobacco use (p=0.784), accompanying disease (p=0.437) and excessive daytime sleepiness scale score.

**Conclusion:**

In our study, patients who had a complaint of sleep disorder before and who were previously admitted to a health center for this problem, were also found to suffer from the same problem during pregnancy. Treatment of sleep disorders in preconception period for women planning pregnancy is important in terms of mother and the baby’s health. Pregnant women should be informed about factors reducing sleep quality during pregnancy.

## INTRODUCTION

Sleeping constitutes about 1/3 of the human life cycle which is an indispensable basic daily activity and affects the life quality and health of the individuals with its physiological, psychological and social dimensions^(1,2)^. Sleepiness is defined as impulse or tendency which directs an individual to sleep^(3,4)^. Sleeping is one of the basic physiological need of human beings which is essential for both physical and psychological health. Healthy adults should fall asleep within 5-10 minutes after closing the light, and at least 7 hours of sleeping activity is needed^(5,6)^. However, individuals’ sleeping needs vary depending on the age, gender, nutrition, activity, health status, environment, individual characteristics and genetic inheritance^(2,7,8)^. Pregnancy is one of the most important events in a woman’s life. Although having a baby is special and meaningful for every woman after a gestation period, women may experience some physical and psychological ailments due to the changes they face during pregnancy^(9,10,11)^. Hormonal and physical changes which occur during pregnancy period cause significant changes in sleep pattern and sleep quality. Besides, the changes in body position and abdominal size during pregnancy period is one of the causative factors of sleep disorders^(12)^. Enhancing the sleep quality and minimizing the sleep problems play important role for a healthy pregnancy period of a mother. According to the Turkish Ministry of Health, General Directorate of Mother-Child Health and Family Planning (MoH, MCH/FP), about 2 million pregnancies occur each year in our country. However, there are a few studies regarding the sleep quality and sleep problems of pregnant women.

Thus, the aim of the present study was to determine the general sleepiness level and factors affecting sleep quality in pregnant women who were admitted to the Obstetrics and Gynecology Clinic at Kahramanmaraş Sütçü Imam University Faculty of Medicine Research and Training Hospital.

## MATERIALS AND METHODS

The study was held between the dates of January 1, 2014 and March 1, 2014 among 100 pregnant women who were admitted to the Obstetrics and Gynecology Clinic at Kahramanmaraş Sütçü Imam University Faculty of Medicine, Research and Training Hospital for any reason. A written informed consent was taken from each of the study participants and questionnaires were applied to all participants with face to face interview technique. The study was approved by the local ethics committee.

Sociodemographic characteristics of the patients and pregnancy-related questions (age, weight, height, total number of pregnancies, sequence number of the current pregnancy, the number of miscarriage, stillbirth, abortion, number of alive children, gestational age, education level, occupation, place of residence, number of individuals in the family, smoking and Maras powder usage, the presence of concomitant diseases, exercise status, admittance to hospital due to sleep disorders) were taken as independent variables.

Maras powder (Nicotiana rustica L); a type of smokeless tobacco called “Crazy Tobacco” (Nicotiana rustica L), which is produced by mixing powder of tobacco with the ash of oak, walnut or vine. It is usually used to quit smoking, however, leads to addiction to itself.

For the collection of data, the Epworth Sleepiness Scale (ESS) and sociodemographic form were used. Statistical analysis was done by using SPSS 20.0 package program. In the data analysis, frequency and standard deviation values were determined. The suitability of variables to normal distribution was analyzed by the Kolmogorov-Smirnov test. To demonstrate the differences between the two groups, chi-square and t-tests were used. A p value of less than 0.05 was considered statistically significant.

The ESS is a simple and self-reporting scale. It questions the general daytime sleepiness level of the individual. Its aim is to evaluate the chance of falling asleep or sleepiness in eight different daily life conditions (sitting and reading, watching TV, sitting, inactive in a public place (e.g. a theater or a meeting), as a passenger in a car for an hour without a break, l ying down to rest in the afternoon when circumstances permit, sitting and talking to someone, sitting quietly after lunch without alcohol, in a car while stopped for a few minutes in traffic). It is an 8-point scale which is simple, easy to understand, and the validity and reliability is proven(13). In our country, ESS reliability and validity studies was conducted by Ağargün and colleagues^(14)^.

## RESULTS

A total of 100 pregnant women with a mean age of 27.97 years (min=16, max=42) were included in the study. 74% of the study participants were living in city center, and the rest of them in countryside, towns and villages. The education status of the participants was as follows: 3% illiterate, 53% primary school graduates, 28% secondary school graduates, and 12% high school graduates. 94% of pregnant women stated that they were non-smoking and the rest 6% stated that they were smokers. In addition, 8% of them were Maras powder users. 6% were previously admitted to hospital due to sleep disorders. 9% of the participants stated to have stillbirth. 25%-to have curettage. 87%-not to do regular exercise and 13% exercising regularly. All sociodemographic findings of the study participants are presented in Table 1, 2, and 3.

No significant changes were found among individuals with regard to exercising regularly, Maras powder usage and having concomitant diseases. The average score of excessive sleepiness of pregnant women was found to be 4.56 (min: 0.00 max: 15). When the patients were compared in terms of sleepiness scale scores, there was no significant differences between the individuals doing regular exercise and not doing regular exercise (p=0.137), between cigarette and Maras powder users and non-users (p=0.965), and between those with concomitant diseases and disease free individuals (p=0.437). A significant association was determined between those who previously admitted to hospital due to sleep disorders and who do not (p=0.006).

## DISCUSSION

Sleeping is one of the most important needs of healthy living. It plays a key role in the growth, development, learning and relaxing activities of humans and is a period that allows people prepare healthy to the next day^(15)^. Sleep disorders are type of discomfort that may occur at every age due to organic, psychiatric or psychological reasons and spread over a wide range disorders from narcolepsy to restless legs syndrome^(3)^. Sleep problem is an indicator of our unconscious responses to the changes in the internal or external conditions. Sleep problems are common in the community. Among them insomnia, difficulty in falling asleep or maintaining asleep, excessive sleep and excessive daytime sleepiness, behavioral disturbances and sleep breathing disorders during sleep are the most commonly seen problems^(16)^. Excessive daytime sleepiness is not a disease or a disorder. This may be a symptom of a sleep disorder or other illnesses. Excessive daytime sleepiness is a major clinic and public health concern^(16,17)^.

Especially in the pregnancy period, in which physical, mechanical, hormonal, emotional changes and new experiences are common in the women’s life, sleeping activities may be affected. Hormonal and physical changes that occur during pregnancy cause significant changes in sleep and sleep quality. Sleep patterns and sleep quality in pregnant women are disturbed by the problems, such as increasing abdominal discomfort as a result of the pressure to the diaphragm made by growing fetus, nocturia, back pain, leg cramps, hormonal changes due to increased progesterone and estrogen levels and restless leg syndrome^(16,18,19)^.

Although sleep disorders have been reported in postpartum period, the number of studies related to sleeping disorders during pregnancy is very limited. Şenol et al., in their study among 300 adolescents, found average ESS score as 4.61±3.87(20). They indicated that 9% of the adolescents complain about excessive sleepiness. It has been reported that 28.2% of children in India, 42.98% of children in Iran, and 50% of children in London need to sleep during daytime^(16,18,19)^, however, the prevalence of sleep disorders in pregnant women is unknown.

The effect of exercise on sleeping has been investigated for over thirty years. There is a contradiction among the results of studies in this field. Some studies^(21,22,23,24)^ reported that regular daily exercise affects sleeping activities positively. On the other hand, some studies^(25,27)^ reported contrary results. In our study, there was no significant difference in sleepiness scores between pregnant women who do regular exercise and who do not.

It is known that cigarettes have stimulating effects due to their nicotine content. Thus, smoking, especially smoking before falling asleep, is known to affect sleep quality adversely. Previous studies have reported that smokers’ sleep quality is poorer than non-smokers^(28,29,30,31)^. In our study, no significant differences were found between the cigarette smoking pregnant women and non-smoking pregnant women. In a study on college students, Saygılı et al. demonstrated that chronic concomitant diseases adversely affect sleep quality^(32)^. Where as in our study, no significant association was found between the concomitant diseases and excessive daytime sleepiness.

In the present study, the average ESS was determined as 4.56. Also, no significant differences were determined between the pregnant women who do regular exercise and who do not, between the cigarette and Maras powder users and non-users and between those with concomitant diseases and disease-free pregnant women.

Sleeping disorder is one of the most frequently seen public health problems that may cause life-threatening accidents, decreased labor productivity and psychosocial functioning impairment.

Accidents at home and outside home which pregnant women suffering from the daytime sleepiness can encounter, possess a risk to both pregnant women and the fetus. Therefore, sleepiness situations of pregnant women should be taken into account and necessary recommendations and measures should be shared with pregnant women. Entire health care team have important roles, especially nurses and midwives, in reducing the problems experienced by pregnant mothers and indirectly enhancing the public health.

## Figures and Tables

**Table 1:**
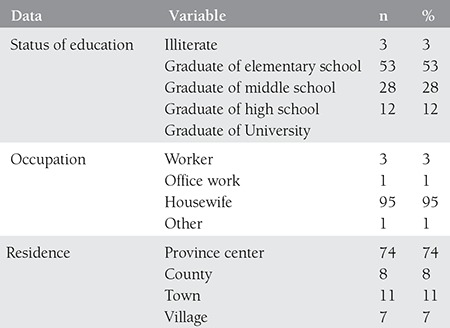
Sociodemographic data of participants

**Table 2:**
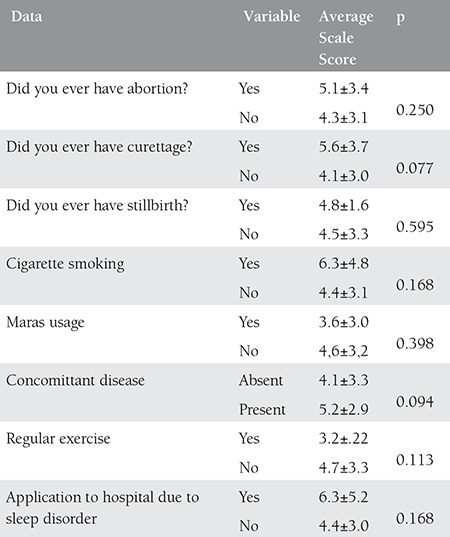
Sociodemographic data

**Table 3:**
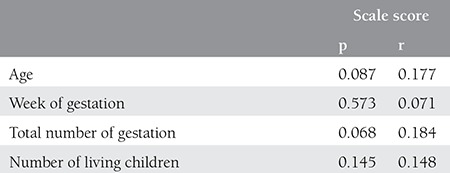
Correlation among the age, week of gestation, total number of gestation and number of living children with regard to average scale scores of pregnant woman
